# Stop Smoking Practitioners’ understanding of e-cigarettes’ use and efficacy with particular reference to vapers’ socioeconomic status

**DOI:** 10.1017/jsc.2018.9

**Published:** 2019-03

**Authors:** Rosemary Hiscock, Deborah Arnott, Martin Dockrell, Louise Ross, Andy McEwen

**Affiliations:** 1 Department for Health, University of Bath, Bath, UK; 2 UK Centre for Tobacco and Alcohol Studies (UKCTAS), Nottingham, UK; 3 Action on Smoking and Health (ASH), London, UK; 4 Public Health England, London, UK; 5 Stop Smoking Service Leicester City Council, Leicester, UK; 6 National Centre for Smoking Cessation and Training (NCSCT), Dorchester, UK

## Abstract

**Introduction:** We have undertaken four online surveys of Stop Smoking Service (SSS) practitioners in England, between 2011 and 2016, in order to enhance our understanding of e-cigarettes: a fast moving new phenomenon. It is important to understand whether e-cigarettes can ameliorate or exacerbate health inequalities given that smoking is one of the most serious causes of excessive mortality and morbidity among disadvantaged groups globally.

**Aims:** To update findings of previous surveys and examine socioeconomic status differences in e-cigarette use and efficacy.

**Methods:** Analysis was undertaken of electronic surveys, particularly, the most recent 2016 survey (*n* = 514) and 2015/16 SSS client routine monitoring data.

**Results:** SSS practitioners were becoming more positive about e-cigarettes: 42% agreed that e-cigarettes were a good thing compared with 15% in 2011. Reported use of e-cigarettes among SSS clients was low (about 3%) despite higher quit rates (63% of clients reported being quit at four week follow-up, compared with 51% overall). Where socioeconomic differences in e-cigarettes’ efficacy for quitting were identified, affluent and working smokers were advantaged.

**Conclusions:** Low use of e-cigarettes by clients and practitioner opinions suggest that further education of SSS staff is needed if they are to adopt the current service recommendations about e-cigarettes.

## Introduction

E-cigarettes, which use battery power to create an inhaled aerosol, were first introduced into the UK towards the end of the first decade of the twenty first century (Public Health England (PHE), 2015; Tobacco Advisory Group of the Royal College of Physicians, 2016). After a period of rapid growth from 2011 to 2013, use has been relatively stable (PHE, 2015; West, Beard, & Brown, 2017); although concurrently use among ex-smokers has grown (Action for Smoking and Health (ASH), 2016b). About 14% of smokers used e-cigarettes in 2015 (UK Office for National Statistics (ONS), 2017a) corresponding to 5.4% of all adults in April 2016 (note that use among non-smokers is minimal) (West et al., 2017). Half of the current smokers in the UK had tried e-cigarettes by 2015 (ONS, 2017a). E-cigarettes can help smokers quit or cut down cigarette use (PHE, 2015) and use for about six months is associated with lower levels of carcinogens than smoking (Shahab et al., 2017). However, e-cigarettes are too new for long-term benefits or harms to be assessed (PHE, 2015).

### Stop Smoking Services and E-cigarettes

In 1999, the UK National Health Service (NHS) set up local Stop Smoking Services (SSS) to help smokers to quit and reduce inequalities in health via the provision of pharmacotherapy and behavioural support to smokers making a quit attempt (McNeill, Raw, Whybrow, & Bailey, 2005). SSS practitioners in England meet a large number of smokers wishing to quit (3,82,500 smokers were treated in 2015–16 (NHS Digital, 2016)) and so are well placed to observe trends in use of e-cigarettes among this population. We have therefore conducted a series of surveys of practitioners’ views on their clients’ use of e-cigarettes in 2011, 2013 and 2014 (Hiscock et al., 2015, 2014) to enhance our understanding of a fast moving new phenomenon.

The SSS are able to prescribe e-cigarettes if the following conditions are met: the e-cigarettes are licensed, commercially available and the local clinical commissioning group, through which local finance decisions are made about government funded health services, agrees to pay for them (Cancer Research UK (CRUK), 2016). At the time of writing, these conditions have never been met so no local SSS are prescribing e-cigarettes. Nevertheless, the National Centre for Smoking Cessation Training (NCSCT) for England and Wales recommends that SSS ‘be open to e-cigarette use in people keen to try them; especially in those that have tried, but not succeeded, in stopping smoking with the use of licensed stop smoking medicines ’(NCSCT, 2014) and NICE guidelines, currently out for consultation and due for publication in March 2018, currently include a recommendation that clients are offered advice on use of e-cigarettes (NICE, 2017). However, in 2014, some local services were not embracing e-cigarettes (Hiscock et al., 2014). Use of the SSS has been declining since 2012, whilst e-cigarette use has become more common but this decline cannot be attributed solely to e-cigarettes (Beard, West, Michie, & Brown, 2016; CRUK, 2016).

### Socioeconomic Status and E-cigarettes

Disadvantaged groups globally, aged between 35 and 69, are much more likely to die from smoking (David, Esson, Perucic, & Fitzpatrick, 2010). Tobacco is responsible for about half the socioeconomic status (SES) difference in these death rates. (Jha et al., 2006). International reviews of studies of SES and e-cigarette use, however, have either concluded that use is increased among high SES groups or that evidence is conflicting (Hartwell, Thomas, Egan, Gilmore, & Petticrew, 2016; PHE, 2015). UK samples suggest more use among higher SES groups (Brown et al., 2014; West et al., 2017). Thus, SES differences between e-cigarette users are not yet clearly established but need to be understood to reduce smoking-related disease.

### Study Objectives

In 2016, in order to continue to understand new developments in the use of e-cigarettes, we conducted a survey of SSS practitioners with a particular focus on SES as such smokers bear the largest burden of disease. The objectives of this paper are to increase the understanding of e-cigarette use and efficacy by comparing responses to the new survey of SSS practitioners with our three previous surveys of SSS practitioners opinions on e-cigarettes and analysis of SSS routine monitoring data in 2016 with particular reference to:

(1) Updating findings from previous surveys.
(2) Exploring SES differences in SSS clients who are vaping.

## Methodology

Two English data sources were analysed for this study. The first comprised of electronic surveys of SSS practitioners. The second was the routine monitoring data collected by the SSS practitioners and collated by NHS Digital (2016). SPSS version 22 and Microsoft Excel 2013 were used for analysis.

### The 2016 electronic Survey of SSS Practitioners

A link to a Survey Monkey (2017) questionnaire was distributed to SSS practitioners via the NCSCT e-bulletin, which is distributed via email. The survey was open between 23 September and 19 November 2016. There were 831 (18%) responses from the estimated 4,724 active practitioners on the database (Brose, 2017 Personal Communication). Sixty-one practitioners (7%) came from outside England and so were excluded from the survey leaving 769 practitioners. Of these, 514 (67%) completed the questionnaire and were included in the analysis.

#### Geographical response

To enable allocation to government office region (GOR) (ONS, 2012), practitioners were asked to state the Local Authority (NHS, 2017) in which they worked. Practitioner distribution by GOR and corresponding regional mid-2015 population estimates (ONS, 2016) were tabulated.

#### Practitioner experience and endorsement of e-cigarettes

Questions on practitioner experience of e-cigarettes were repeated from previous surveys (Hiscock et al., 2015, 2014). Practitioners were asked how numbers of enquiries from SSS clients about e-cigarettes compared to the previous year and to indicate whether practitioners recommend e-cigarettes to all clients, no clients or specific clients groups. Additionally, practitioners were asked the extent to which they agreed with a series of statements using five point Likert scales. For tabulation ‘agree’ and ‘strongly agree’ options were merged as were ‘neither agree not disagree’, ‘disagree’ and ‘strongly disagree’. The statements were: ‘e-cigarettes are a good thing’, ‘over the past 12 months I've become less positive about e-cigarettes’, ‘e-cigarettes ‘normalise’ cigarette smoking’, ‘e-cigarettes ‘de-normalise’ cigarette smoking’. Chi-square tests compared results from the 2016 survey with published results from the surveys conducted in 2014 (*n* = 1,801), 2013 (*n* = 705) and 2011 (*n* = 587) (Hiscock et al., 2015, 2014).

#### Practitioner opinions on e-cigarette differences by SES

Practitioners were asked whether, in their opinion, particular SES groups were more likely to use e-cigarettes or less likely to quit using e-cigarettes. High SES clients (clients with professional, managerial and intermediate occupations (PMI)) were compared with two low SES client groups: first, clients with routine and manual occupations (R&M) and second, clients who were unemployed and permanently sick. Note that the three groups were based on SES categories from NS-SEC (ONS, 2008), which practitioners use to classify clients for routine monitoring returns so that they would be familiar with classifying clients in this way. Use and efficacy among the three SES groups were compared using Cochran's Q-tests.

In addition to statistical analysis, it is also useful to analyse diversity of opinion using a more qualitative methodology (Jansen, 2010). Therefore, as well as answering closed questions, practitioners were asked to comment on the reasons they believed lay behind socioeconomic differences or lack of differences in successful e-cigarette use. Unidimensional descriptive qualitative analysis of survey data was used (Jansen, 2010). Downward coding (Jansen, 2010) specified diversity within comments supporting and opposing SES differences. Thus, supporting and opposing comments were divided by one researcher into major themes and sub themes. This approach was similar to our previous analyses of comments in the SSS practitioner surveys (Hiscock et al., 2014). In this analysis, focusing on SES differences, the analysis was informed by previous work on SES and health (Hiscock, Schieberle, Li, Gari, & Grimalt, 2015) and SES and smoking (Hiscock, Bauld, Amos, Fidler, & Munafo, 2012; Hiscock, Bauld, & Judge, 2010). Subthemes were used to write a short description of each major theme. As a very informal validation (Komori & Christine, 2013), the number and percentage of participants who endorsed each theme was tabulated.

### SSS Routine Monitoring Data

SSS statistics by Local Authority area (April 2015 to March 2016) were downloaded from NHS Digital (2016). For each Local Authority, the following were collated: number of clients who set a date for quitting smoking with a practitioner (a quit date), number of clients who self-reported that they had quit overall and number of various subgroups who self-reported that they had quit. Self-report quit was defined as a client stating they had not smoked when followed up 28 days (−3 or +14 days) after the quit date (NCSCT, 2014) (note that smoking was allowed for the first 14 days of a quit attempt). In addition, the number of self-reported quitters with a carbon monoxide (CO) reading of less than 10 parts per million (ppm) in each Local Authority was downloaded (CO validated quit (NCSCT, 2014)). CO validated quit rates for subgroups were not consistently available. The subgroups were pharmacotherapy (single Nicotine Replacement Therapy (NRT), combination NRT, buproprion (Zyban), varenicline (Champix), NRT and Champix/Zyban, consecutively, NRT and Champix/Zyban concurrently, licensed medication and e-cigarettes, only e-cigarettes, no medication or e-cigarettes, quitting aid unknown), NS-SEC (professional and managerial, intermediate, routine and manual, student, caring for home/family, unemployed, prisoners, retired, long-term sick and disabled, SES unknown).

The median Local Authority self-report and CO validated quit rate and the range of quit rates were calculated. Median self-report quit rates for subgroups were calculated. Medians were calculated as some characteristics were not normally distributed. The two measures of e-cigarette used were summed in order to create a variable measuring percentage of using e-cigarettes (with or without other pharmacotherapy). Non-parametric correlations (Spearman's rho) were computed between the subgroups and six outcomes: % self-report quit, % CO validated quit, % using e-cigarettes, % using e-cigarettes and licensed pharmacotherapy and % using just e-cigarettes.

## Results

### Survey of SSS Practitioners

#### Geographical response

Practitioners responded from all English regions (Table 1). There were 2% practitioners whose region could not be allocated usually because they did not work directly for a local authority and their response did not give a geographical location.

**Table 1:** Practitioner responses by geographical region in which employed

	*N*	%	% English Population
North East	25	4.9	5%
North West	51	9.9	13%
Yorkshire and Humberside	40	7.8	10%
East Midlands	46	8.9	9%
West Midlands	47	9.1	10%
East of England	57	11.1	11%
London	94	18.3	16%
South East	82	16.0	16%
South West	61	11.9	10%
Not provided	11	2.1	
Total	514	10.0	

#### Comparison of the 2016 survey with previous surveys

Practitioners were significantly more positive towards e-cigarettes in 2016 than in the other surveys (42% (2016), 24% (2014), 26% (2013) and 15% (2011) strongly agreed or agreed that e-cigarettes are a good thing, *p* < .001). Reported growth in the number of client enquiries over the previous year was significantly higher in 2013 than 2016 (*p* < .001) but there was no difference between 2016 and 2011 (Table 2). In 2016, compared to 2014, practitioners were significantly more likely to recommend e-cigarettes to all clients (15% vs 5% *p* < .001), current users (23% vs 18% *p* < .05), clients cutting down (18% vs 12% *p* < .001) (including cutting down to stop (18% vs 12% *p* < .001)) and clients who had made many quit attempts (30% vs 20% *p* < .001). Moreover, there were significantly fewer practitioners who reported not recommending e-cigarettes to any clients (32% in 2016 vs 56% in 2014, *p* < .001).

**Table 2:** Stop smoking practitioners' views of e-cigarettes 2016 compared with previous surveys

	2016			2014			2013			2011		
	*n*	*N*	%	*n*	*N*	%	*n*	*N*	%	*n*	*N*	%
*Agree/strongly agree (vs neutral, disagree and strongly disagree)*												
E-cigarettes are a good thing	218	514	42.4	429	1,758	24.4***	182	703	25.9***	87	588	14.8***
Over the past 12 months I've become less positive about e-cigarettes	72	514	14.0	495	1,762	28.1***						
E-cigarettes ‘normalise’ cigarette smoking	182	514	35.4	950	1,763	53.9***						
E-cigarettes ‘de-normalise’ cigarette smoking	77	514	15.0	190	1,769	10.8**						
*More clients asking about e-cigarettes compared to one year ago (vs same or fewer clients asking)*	338	514	65.8				607	669	90.7***	338	526	64.3
*Client groups to which e-cigarettes would be recommended:*												
All of my clients	75	514	14.6	82	1,745	4.7***						
Those clients who have tried and failed to quit many times	154	514	30.0	355	1,757	20.2***						
Clients who are already using e-cigarettes	119	514	23.2	324	1,762	18.4*						
Those clients wishing to cut down but not stop smoking	94	514	18.3	215	1,762	12.2***						
Clients who wish to cut down before they stop	91	514	17.7	204	1,759	11.6***						
Clients wanting to use e-cigarettes at times when they cannot smoke (temporary abstinence)	74	514	14.4	212	1,767	12.0						
More dependent smokers	74	514	14.4	199	1,761	11.3^+^						
None of my clients	162	514	31.5	983	1,762	55.8***						

***p < .001, **p < .01, *p < .05, ^+^*p* < .10 (chi-square tests compared to 2016).

#### SES

Practitioners most often stated that clients with R&M occupations were most likely use e-cigarettes (34% vs 23% clients with PMI and 24% unemployed and long-term sick and disabled) (Table 3). Unemployed and long-term sick and disabled clients were considered to be the least likely to quit using e-cigarettes (20% vs 7% R&M and 5% PMI). The lowest and highest SES groups significantly differed (*p* < .001). However, over 50% of respondents indicated that there was no difference or that that they did not know if there was a difference in use and efficacy.

**Table 3:** Practitioner opinions of SES differences in e-cigarette use and efficacy (2016) (*N* = 514)

	Use E-cigarettes		Least Likely to Quit with E-cigarettes	
	*N*	%	*N*	%
Professional, managerial and Intermediate	117	22.8	23	4.5
Routine and manual	176	34.2	35	6.8
Unemployed, long-term sick and disabled	122	23.7	105	20.4
Cochran's *Q* test		*p* < .001		*p* < .001
No difference	163	31.7	135	26.3
Do not know	110	21.4	241	46.9

When asked to comment on why they thought there were (or were no) SES differences, 57% did not leave a comment, 28% left comments suggesting that they did not discern a difference and 16% left comments suggesting that there was an SES difference.

Eight themes, or reasons for SES differences in successful e-cigarette use, were identified from comments identifying an SES difference (Table 4). Of the sample, 6.4% said they thought that SES differences were due to low income – inability to pay for the best quality e-cigarettes and accompanying paraphernalia meant that low income people were less likely to quit with e-cigarettes. Differences in cognition were suggested by 5% practitioners leading to reduced quitting among low SES groups. These differences included poorer information collation and understanding of information. Additionally, some practitioners suggested that low SES smokers sometimes had less willpower and motivation to quit smoking. Some practitioners’ comments implied that there appeared to be a cultural difference: low SES groups were suggested to be happier to vape long-term either with or without combustible cigarettes: ‘lower socio-economic groups more likely to replace smoking with E-Cigarettes and also to concurrently smoke rather than use e-cig[arette]s as a cessation aid’.

**Table 4:** Practitioner suggested reasons for SES differences in successful use of e-cigarettes (*N* = 514)

	*N*	%
Low income (problems affording e-cigarettes in particular the best quality e-cigarettes, starter packs, spare parts, however, ability to reuse may be appealing)	33	6.4
Cognitive differences (poor information collation on the best e-cigarettes and correct use (more likely to rely on peers and tabloid scare stories), lack of interest in the future and low self-esteem may be reflected in lower motivation to quit and willpower, low SES appear happier to vape and smoke or replace smoking with vaping instead of using e-cigarettes to quit smoking)	27	5.3
Social differences (low SES peers are more likely to smoke or vape compared with those with professional and managerial jobs and those with such jobs are seen as role models so might feel more pressure to successfully quit)	15	2.9
Time activity (those not working have more spare time at home where e-cigarettes are not banned, those in routine and manual jobs have breaks where many vape or smoke, however, stress in prof/managerial jobs or routine and manual jobs might reduce quit success)	7	1.4
*Reasons suggested by <1% practitioners*		
Low SES have poor outcomes generally for quitting smoking whatever quitting aids used due to multiple issues		
Dependency (low SES are more dependent on nicotine and e-cigarettes might exacerbate this due to superior nicotine delivery)		
Availability (e-cigarettes are less available than combustible cigarettes so low SES may be less likely to come across them but vape shops are more likely to be found in low SES areas)		
SSS lack of engagement with e-cigarettes (e-cigarette users are less likely to engage with SSS so get less help to quit, partly because SSS are ambivalent about their use)		

This difference in long-term vaping could arise from the third theme social differences: it was suggested that clients with professional and managerial occupations were more likely to be seen as role models and thus be under more pressure to give up tobacco completely; in addition vaping may be seen as ‘less socially acceptable’ among PMI social groups, whereas among other social groups ‘nicotine addiction [is] more accepted in general’.

The fourth theme was time activity. An SSS practitioner suggested that R&M occupations’ working hours were more often geared towards smoking (and now vaping) breaks. Furthermore, workplace restrictions on vaping were not experienced by people not in the workplace such as the unemployed and long-term sick: ‘smoke more as they are at home & so have more time on [their] hands. A professional will be working & less likely to be able to smoke the e-cig[arette] when they want’. Note this practitioner was using the term ‘smoke’ rather than ‘vape’ to refer to active use of e-cigarettes.

Other themes were mentioned by less than 1% practitioners each: low SES poorer outcomes generally might extend to attempting to quit via e-cigarettes; low SES higher dependence may lead to e-cigarettes being harder to give up and availability: this could work in either direction– low SES groups, with poorer information gathering skills, may not come across e-cigarettes but on the other hand, one practitioner suggested that vape shops were more often located in low income areas.

In the opinion of practitioners, there were three main reasons to explain a lack of association between SES and e-cigarette use (Table 5). The first theme was that SES differences were not as a result of SES itself but of individual differences, willpower, dependency and lack of information campaigns: for example, ‘I think it depended on the level of dependence rather than socio-economic group’. Thus, these reasons were the same as some of the themes that suggested that there were SES differences. The latter two themes disagreed with two of the themes suggesting that there was a difference: first, e-cigarettes were argued to be ubiquitous and available to all and second, e-cigarettes were argued to be cheap enough for all to access.

**Table 5:** Reasons for classification as ‘no discernible difference’ (*N* = 514)

	*N*	%
*No difference*	61	12.2
*Socioeconomic status not important:* Social class itself is not important (or is less important than individual differences for example in willpower or dependency). Informational campaigns can spread information to all social classes	26	5.2
*Ubiquitous:* All groups are exposed to e-cigarettes and can acquire them. All groups use them (they are trendy) and their success is not different	12	2.4
*Cost*: They are cheap enough for everyone to use or, at the very least, there are cheap options	8	1.6
No reason given	15	3.0
*Unsure whether or not there is a difference*	44	8.8
*Insufficient client experience:* Unaware of clients SES (for example works with teenagers), or all clients from one SES group, insufficient vapers among clients or does not work directly with clients	22	4.2
*No analysis of this* either by the practitioner themselves or from the research community	10	2.0
No reason given	14	2.8
*Made a general comment not linked to SES*	16	3.2

*Note:* Comments could relate to more than one theme.

Some practitioners indicated that they did not know whether there were SES differences in e-cigarette use often either due to their client group or due to insufficient analysis (either by themselves or by the research community).

### SSS Routine Monitoring Data 2015–16 Financial Year

There were 152 Local Authority areas but two submitted no data (Table 6). Only 3% clients on average were reported to be using e-cigarettes as part of their quit attempt (and the Local Authorities ranged between 0% and 49%). Overall 51% (range 28–82%) of clients self-reported as quit and 38% (range 12–59%) were CO validated as quit four weeks after their quit date; however, there was a large range between Local Authorities.

**Table 6:** Local Authority distribution and quit rates overall and by pharmacotherapy and SES and overall e-cigarette use

	%				% Self-report Quit			
	N*	Median	Min	Max	*N*	Median	Min	Max
% self-report quit	150	50.9	28.2	82.8	150	50.9	28.2	82.8
% CO validated quit	150	37.5	11.9	59.4				
% e-cigarettes	150	3.1	.0	49.4	130	63.1	.0	100.0
Pharmacotherapy								
% single NRT	150	22.1	.0	89.0	148	48.3	23.1	88.5
% combination NRT	150	34.2	.0	82.4	137	45.9	23.3	82.7
% zyban	150	.3	.0	4.1	19	53.8	15.4	66.7
% champix	150	24.0	1.7	61.3	149	59.2	24.1	90.7
% NRT and champix/zyban consecutively	150	1.5	.0	17.4	78	48.3	18.0	87.0
% licensed and e-cigarettes concurrently	150	1.0	.0	24.9	77	60.7	25.0	89.3
% licensed and e- cigarettes consecutively	150	.3	.0	1.7	28	65.2	36.4	89.7
% only e-cigarettes	150	.3	.0	22.0	34	67.5	5.1	98.6
% no medication or e-cigarettes	150	3.9	.0	49.4	126	56.5	.0	96.3
% quitting aid unknown	150	1.0	.0	51.1	76	28.6	.0	85.2
NS-SEC								
% professional and managerial	150	11.7	2.4	77.3	149	57.8	27.1	89.8
% intermediate	150	7.2	1.7	17.6	147	55.1	23.0	88.5
% routine and manual	150	25.5	6.7	68.4	150	53.2	23.6	86.1
% student	150	3.1	.3	13.3	138	39.6	11.8	85.9
% caring for home/family	150	4.9	.0	10.0	144	48.0	26.5	79.5
% unemployed	150	11.9	2.3	32.0	147	42.5	1.8	85.0
% prisoners	150	.0	.0	38.4	41	42.3	6.3	82.9
% retired	150	12.8	1.3	20.8	148	58.4	34.6	85.9
% long-term sick and disabled	150	7.9	.0	14.7	147	44.7	2.0	83.3
% SES unknown	150	7.2	.0	53.3	116	46.2	12.0	81.7

*N refers to the number of Local Authorities analysed.

The median quit rate was 63% for e-cigarette users. Other pharmacotherapies (NRT and Champix – either alone or in combination) were much more commonly reported as being used than e-cigarettes. Quit rates however were highest for e-cigarette users (although Champix alone (59%) was similar).

A quarter of clients held R&M occupations and just over 10% held PM occupations were retired or unemployed. Quit rates approached 60% among retired and those with PM occupations, whereas the quit rates of clients who were students or had a long-term condition were below 45%.

A higher self-reported Local Authority quit rate (Table 7) was positively and significantly associated with higher levels of clients with PMI and R&M occupations. They were negatively and significantly associated with higher levels of clients who received combination NRT, were unemployed or for those whose SES was unknown. There were significant positive correlations between % clients using Champix and % clients with R&M occupations and the CO validated quit rate. There were significant negative correlations between % missing data on either SES or pharmacotherapy and the CO validated quit rate. There was not a significant relationship between level of e-cigarette use in an Local Authority and either quit rate.

**Table 7:** LA correlations (Spearman's rho) between quitting and e-cigarette use, SES and pharmacotherapy (*N* = 150)

							% Used E-cigarette		% Used E-cigarette			
	% Self		% CO		% Used		and Other		and Other		% Used	
	Report Quit	*p*	Validated Quit	*p*	E-cigarette	*p*	Medication Concurrently	*p*	Medication Consecutively	*p*	E-cigarette Only	p
% self-report quit	1.000	<.001	.604**	<.001	−0.041	.619	−0.018	.824	0.015	.860	0.013	.876
% CO validated quit	.604**	<.001	1.000	<.001	−0.009	.914	0.031	.709	0.048	.558	−0.060	.462
% used e-cigarette	−0.041	.619	−0.009	.914	1.000	<.001	.904**	<.001	.784**	<.001	.708**	<.001
SES												
% prof and managerial	.213**	.009	0.077	.347	0.021	.799	0.072	.382	0.008	.920	0.000	.999
% intermediate	.169*	.039	0.055	.505	0.057	.488	0.083	.314	0.022	.793	0.115	.162
% routine and manual	.241**	.003	.213**	.009	0.145	.077	0.157	.055	.174*	.033	0.016	.844
% student	0.132	.108	0.096	.240	−.282**	<.001	−.256**	.002	−.262**	.001	−.217**	.008
% home carer	0.059	.476	−0.038	.648	.231**	.004	.214**	.008	.174*	.033	0.116	.159
% unemployed	−.169*	.039	−0.059	.470	−0.086	.294	−0.070	.393	−0.068	.411	−0.110	.181
% prisoners	−0.149	.070	−0.074	.369	.186*	.023	.207*	.011	.188*	.021	0.067	.414
% retired	0.104	.205	0.040	.624	.278**	.001	.244**	.003	.306**	<.001	0.154	.060
% long-term sick and disabled	0.042	.609	0.079	.335	−0.065	.427	−0.037	.651	0.037	.656	−0.136	.097
% SES unknown	−.261**	.001	−.181*	.027	−0.135	.099	−.203*	.013	−.182*	.026	0.085	.298
Pharmacotherapy												
% single NRT	0.066	.422	−0.066	.420	−.285**	<.001	−.355**	<.001	−.374**	<.001	−0.063	.443
% combination NRT	−.190*	.020	0.059	.472	.163*	.046	.228**	.005	.198*	.015	−0.010	.905
% zyban	−0.046	.579	−0.072	.379	0.089	.279	0.041	.619	0.103	.208	0.147	.073
% champix	0.121	.141	.168*	.039	.279**	.001	.324**	<.001	.263**	.001	.189*	.021
% NRT and champix/zyban consecutively	0.120	.144	0.065	.430	.421**	<.001	.506**	<.001	.373**	<.001	.238**	.003
% licensed and e- cigarettes concurrently	−0.018	.824	0.031	.709	.904**	<.001	1.000	<.001	.717**	<.001	.504**	<.001
% licensed and e- cigarettes consecutively	0.015	.860	0.048	.558	.784**	<.001	.717**	<.001	1.000	<.001	.498**	<.001
% only e-cigarettes	0.013	.876	−0.060	.462	.708**	<.001	.504**	<.001	.498**	<.001	1.000	<.001
% no pharmacotherapy	−0.069	.404	−.202*	.013	−0.035	.667	−0.026	.750	−0.033	.693	.173*	.034
% pharmacotherapy unknown	−0.077	.346	−0.122	.136	−.171*	.036	−.223**	.006	−0.041	.622	−0.055	.502

Local Authorities with higher use of e-cigarettes also had significantly higher percentages of clients using Champix or combination NRT and prescription medication mixed with NRT, clients caring for homes and families, prisoners and retired clients. Such Local Authorities had significantly lower levels of clients using single NRT or clients whose use of pharmacotherapy was unknown and students.

## Discussion

Many SSS practitioners in 2016 suggested interest in e-cigarette use among SSS clients was continuing to grow, however, this belief was less common than in the 2014 survey. This may reflect more recent stabilisation in use of e-cigarettes (PHE, 2015; West et al., 2017). SSS practitioners who answered the survey in 2016 had become more positive about e-cigarettes than previously. However, 32% practitioners were never recommending e-cigarettes to clients. Furthermore, e-cigarette use among SSS clients reported in routine monitoring data was still minimal (on average 3% clients in a local authority were using e-cigarettes) despite high quit rates for e-cigarette users (63%) (although conversely high quit rates in a small population could potentially suggest that e-cigarettes are only useful for a small proportion of smokers). Minimal growth in client e-cigarette use had occurred since the time of the 2014 survey where routine monitoring data recorded 2% SSS clients to be using e-cigarettes (Hiscock et al., 2015). This suggests that many practitioners have not taken up 2014 guidelines that SSS should be open to e-cigarette use (NCSCT, 2014). This may reflect e-cigarettes not being prescribed by SSS.

Many practitioners did not identify SES differences in vaping. Some practitioners preferred not to see client differences through the prism of social status but instead identified factors causing SES differences. A number of practitioners suggested that high SES smokers viewed e-cigarettes simply as a quitting aid perhaps influenced by a non-nicotine using peer group and thus e-cigarettes were being used short term as part of a successful quit attempt. Conversely, some practitioners suggested that, lower SES smokers were more likely to develop a lifestyle of vaping long-term and e-cigarette use did not necessarily lead to quitting combustible cigarettes. Long-term e-cigarette use was supported by more nicotine use among peers with established work breaks for nicotine use or being out of the workplace where restrictions on use may apply. The routine monitoring data suggested that some clients who did not experience the work environment in their day to day lives did vape more (home carers, retired and prisoners).

More in depth and theoretical qualitative research is needed to confirm whether this cultural social class difference does exist and thus to formally validated these results. Furthermore, smoking prevalence rates disaggregated by SES need to be monitored to see whether SES differences in smoking prevalence emerge, possibly as a reflection of lower quit rates among low SES e-cigarette users.

E-cigarettes have only been available in the UK for around a decade and significant levels of use less than that so there is limited evidence of long-term use. However, long-term e-cigarette use has been shown to deliver similar reductions in levels of measured carcinogens and toxins relative to smoking only combustible cigarettes as NRT (Shahab et al., 2017) and government regulations are intended to ensure e-cigarette minimum safety and efficacy standards (European Commission, 2014; MHRA, 2016). Vaping cultures have previously been identified among youth (Yule & Tinson, 2017). A study in a disadvantaged area of England found a culture of vaping only among young men (Thirlway, 2016) who had time and resources to find e-cigarettes that suited them.

## Limitations

E-cigarette users themselves did not take part in this study. The information about e-cigarette use and effectiveness was gathered second hand via a survey of the opinions of SSS practitioners and analysis of SSS routine monitoring data. SSS practitioners see around 6% of the 6.3 million smokers in England (ONS, 2017b), so practitioners are not be able to report on the behaviour of most smokers and smokers who approach the services may not be representative.

As far as we are aware, it is unknown whether practitioner sociodemographic characteristics influence opinions of e-cigarettes although in a previous study, we found that managers and commissioners were more open to e-cigarettes than client facing practitioners (Hiscock et al., 2015). Future studies may like to focus on practitioners’ sociodemographic characteristics.

There appears to be large numbers of inactive practitioners on the NCSCT mailing list, perhaps reflecting practitioners leaving due to reduced service use and government cuts (Buck, 2017; Hunt, 2016). Thus, the size of the sampling frame is estimated. It is likely that the response rate was low (under 20%). However, responding practitioners were spread throughout the regions of England and, furthermore, we also included the routine monitoring data: a census of all clients who set a quit data with the SSS. The routine monitoring data was not collected for the purposes of this analysis. Thus, our analysis was restricted to local authority level data rather than individual client level data.

Two months after the survey was in the field a tobacco company introduced a heated tobacco product for the first time to the UK market and other such products are likely to be launched in future (ASH, 2016a). Such products are likely to compromise health but be less risky than conventional cigarettes; comparison with e-cigarettes is as yet unavailable (Committee on Toxicity, 2017). Future surveys may need to introduce assessments of these products if they become widely used.

## Summary

SSS practitioners who respond to the survey are becoming more positive about e-cigarettes. Local SSS report that the minority of SSS clients who do use e-cigarettes are usually one of the groups that have the highest quit rates. This may suggest that the positive benefits of e-cigarettes as an aid to quitting should be promoted among SSS practitioners. A minority of SSS practitioners suggested that e-cigarettes would be more likely to lead to quitting among higher SES smokers but most did not discern a difference.
